# Free-standing Reduced Graphene Oxide/Carbon Nanotube Paper for Flexible Sodium-ion Battery Applications

**DOI:** 10.3390/molecules25041014

**Published:** 2020-02-24

**Authors:** Yong Hao, Chunlei Wang

**Affiliations:** 1 Department of Mechanical and Materials Engineering, Florida International University, 10555 W. Flagler St., Miami, FL 33174, USA; yhao004@fiu.edu; 2 Center for the Study of Matter at Extreme Conditions, Florida International University, University Park, Miami, FL 33199, USA

**Keywords:** reduced graphene oxide/carbon nanotube (rGO/CNT), free-standing, anode, sodium-ion batteries, flexible

## Abstract

We propose a flexible, binder-free and free-standing carbonaceous paper fabricated via electrostatic spray deposition using reduced graphene oxide/carbon nanotube (rGO/CNT) as a promising electrode material for flexible sodium-ion batteries (NIBs). The as-prepared rGO/CNT paper exhibits a three-dimensional (3D) layered structure by employing rGO as conductive frameworks to provide sodium-storage active sites and CNT as spacer to increase rGO interlayer distance and benefit the diffusion kinetics of sodium ions. Consequently, the rGO/CNT paper delivers an enhanced sodium ion storage capacity of 166.8 mAh g^−1^ at 50 mA g^−1^, retaining an average capacity of 101.4 mAh g^−1^ when current density sets back 100 mA g^−1^ after cycling at various current rates. An average capacity of 50 mAh g^−1^ at 200 mA g^−1^ was stabilized when cycling up to 300 cycles. The well-maintained electrochemical performance of free-standing rGO/CNT paper is due to the well-established hybrid 3D nanostructures, which demonstrates our carbon based material fabricated by a facile approach can be applied as one of the high-performance and low-cost electrode materials for applications in flexible energy storage devices.

## 1. Introduction

Although lithium-ion batteries have dominated the global electrochemical energy storage market in portable electronics for the past three decades, large scale applications such as electric vehicles and renewable energy integrations call for alternatives beyond Li-ion energy storage systems [1,2]. Sodium-ion batteries (NIBs) have drawn enormous attention owing to the inexhaustible Na resources and the similar electrochemical reaction mechanism to lithium metal [3,4,5]. However, there are challenges that need to be tackled before NIBs can be commercialized. One of the challenges is the relatively large size of Na ion (0.102 nm vs. Li ion: 0.059 nm), which could potentially reduce the efficiency of Na ion intercalation and de-intercalation if electrode materials do not exhibit favorable structures [6,7]. Recently various studies have been conducted to identify advanced electrodes with efficient kinetics and high storage capacity for NIBs to improve their electrochemical performance [8,9,10,11,12,13]. Carbonaceous materials with advantages of low-cost, eco-friendliness and high conductivity attract great interest as electrode material candidates for NIBs. Various carbonaceous materials including porous carbon [14,15], hard carbon [16], carbon nanofibers [17,18], graphene [7,19] and carbon nanotubes [20,21] have been studied and found to facilitate the sothiation/desothiation process of Na^+^ into/from the active electrode hosts owing to the large interlayer distance and/or disordered structure. Among them, graphene and CNT are considered as two promising candidates in energy storage systems owing to their unique dimensional structures and beneficial intrinsic properties such as chemical stability, low weight, high mechanical flexibility and excellent electrical conductivity [22,23].

In addition to the electrode material itself, recently, flexible and bendable electronic devices have been urgently required. In order to power these devices, there is a persistent need to build multifunctional energy storage systems that are equally flexible and bendable under deformation while maintaining their electrochemical function [24]. Recently, a lot of research efforts have been focused on designing free-standing and binder-free electrodes with excellent flexibility [25]. For these types of electrodes, the elimination of polymer binder and conductive additive which are the inactive electrode components could reduce the overall mass of the electrode and be beneficial for the improvement of energy density. This also benefits to avoid the detachment at the interface between metal foil current collector and the electrode during charge/discharge process, which is one of the major issues for the flexible batteries due to the bending or folding of the devices [26]. To make the electrode materials flexible while achieving high energy density, different synthesis methods have been used such as hydrothermal, solvothermal, vacuum filtration, electrospinning, etc. [25]. Compared with other methods, the electrospinning technique as a remarkably simple, versatile and controllable method, has been used to generate nanofiber-containing flexible fibrous mats with different composition and thickness [27]. A variety of free-standing carbon fiber anode materials fabricated by electrospinning have been investigated for NIBs [17,28,29,30]. To fabricate the carbon fibers, various polymer solutions are used as precursors to synthesize polymer fibers and followed by subsequent pyrolysis [22]. Free-standing graphene films or graphene-assisted composites have been fabricated by thermal or filtration methods [7,11,31,32]. However, when graphene is individually employed as anode for NIBs, graphene could only deliver a limited storage capacity due to strong preferential stacking orientation of compact graphene nanosheets in the conventionally filtrated graphene in which Na ions cannot sufficiently transport. Therefore, the strategies of heteroatom doping in carbonaceous materials or designing hybrid nanostructures containing graphene have been proposed to avoid the stacking of graphene nanosheets and enhance the overall electrochemical performance of NIBs [33,34]. An et al. demonstrated that fluorine and nitrogen co-doping on graphene could increase the structural defects and expand the interlayer distance, which improved electrochemical performance with a stable capacity of 203 mAh g^−1^ at 50 mA g^−1^ current density after 100 cycles [7]. Besides the doping method, Yan and his colleagues proposed sandwich-like nanocomposites assembled by employing porous carbon with a hierarchical structure on both surfaces of graphene to facilitate diffusion of Na ions. By designing this hybrid structure, the composite exhibited specific capacity of 400 mAh g^−1^ at current density rate of 50 mA g^−1^ in 100th cycle [14]. Liu et al. combined two strategies and prepared a carbonaceous hybrid material with sandwich-like nanostructure by using N-doped carbon coated on graphene sheet. The hybrid material exhibited a large distance of 0.36 nm between interlayers and exhibited a sodium electrochemical battery performance of 336 mAh g^−1^ at 30 mA g^−1^ after 200 cycles [6].

**Figure 1:**
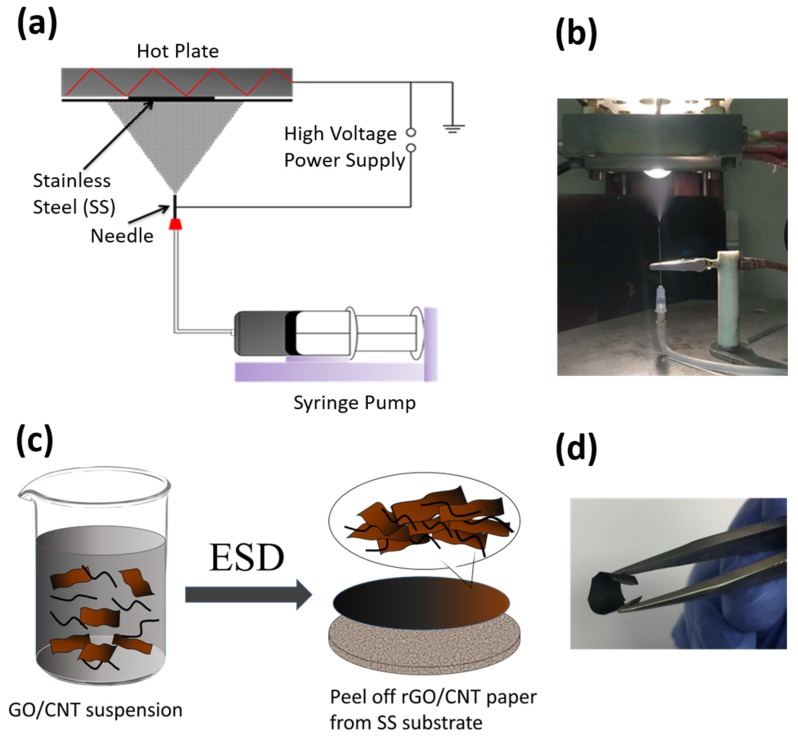
(**a**) Schematic illustration of ESD set-up that was used to fabricate rGO/CNT samples, Digital images of (**b**) shows actual ESD set-up, (**c**) Schematic of rGO/CNT fabrication and (**d**) a digital image of free-standing rGO/CNT paper.

Similar to the electrospinning technique, electrostatic spray deposition (ESD) is an easily-operated and efficient technique to prepare binder-free electrodes for energy storage systems [35,36]. No subsequent heat treatment is needed since the decomposition and reaction directly occur before or after the as-prepared solution/suspension droplets arrive at the heated substrate [37]. Figure 1a,b present a schematic illustration of ESD and an actual setup in the lab, respectively. The electrodes are fabricated as thin films with various morphologies and structures controlled by the ESD experimental parameters, taking the voltage, the flow rate, the substrate temperature for instance. Both dissolved precursors and suspensions are prepared to fabricate different types of oxides, sulfides, carbides, phosphates, carbonaceous materials by the ESD technique [38].

Our previous work has demonstrated that ESD-fabricated reduced graphene oxide/carbon nanotube (rGO/CNT) nanocomposite is a promising electrode candidate for both microsuper-capacitors and Li-ion capacitors [39,40]. In this work, we extend our investigation by using ESD to fabricate free-standing rGO/CNT paper and electrochemically evaluating this material as an anode for NIBs. CNT is employed as a separator to eliminate the stacking phenomenon and expand the interlayer distance of rGO to optimize the Na ion’s transportation pathway. Benefiting from hybrid structure, rGO/CNT paper as a free-standing and flexible anode for NIBs retains a recovered capacity of 101.4 mAh g^−1^ at density rate of 100 mA g^−1^ after cycling at various rates.

## 2. Experimental

### 2.1. Fabrication of Freestanding rGO/CNT Paper

The raw single-layer GO powder and multi-walled carbon nanotubes (MWCNT, outer diameter: 8–15 nm, Length: 10–50 µm) were purchased from Cheaptubes Inc. (Grafton, VT, USA). MWCNTs were first subjected to an acid treatment process to remove the metal catalyst residues and CNTs cut into small segments of 0.2–1 μm. To fabricate one piece of freestanding rGO/CNT paper, 5.4 mg GO and 0.6 mg MWCNT were first dispersed into 20 mL 1,2-propanediol and with sonicated with a probe for 1 h in an ice bath to form a precursor suspension with a concentration of 0.3 mg mL^−1^. Then a syringe pump was used to feed the precursor suspension at a rate of 3 mL h^−1^ into a stainless steel needle. A spacer used in coin cells with a diameter of 14 mm was taken as the substrate. It was placed on a hot plate beforehand and preheated to 300 °C. A voltage of 5–6 kV was applied between the needle and the stainless steel spacer substrate while the ESD process was carried out for 2 h. After deposition, the free-standing rGO/CNT paper was detached from the substrate. The mass loading of the free-standing rGO/CNT paper is about 0.6–0.8 mg. Figure 1c,d show a schematic of the fabrication process and a digital image of as-prepared rGO/CNT paper, respectively.

### 2.2. Characterization and Electrochemical Measurements

X-ray diffraction (XRD) patterns were generated to study the crystal structures of GO, rGO, CNT and rGO/CNT paper by using a D5000 X-ray Diffractometer (Siemens, Munich, Germany) with Cu Kα radiation. The morphology of rGO/CNT paper was studied using a FE6330 field-emission scanning electron microscope (FESEM, JEOL, Tokyo, Japan). Electrochemical measurements were performed with CR2032-type coin cells which were assembled in a glove box with inert argon gas. Coin cells were constructed with free-standing rGO/CNT paper as well as two control samples rGO and CNT as working electrodes, 1 M NaClO_4_ in ethylene carbonate (EC) and dimethyl carbonate (DMC) as electrolyte (*v*/*v* 1:1), Celgard 2400 polypropylene film as a separator and Na foil as both reference and counter electrodes. Cyclic voltammetry (CV) was measured using a VMP3 system (Biologic, Seyssinet-Pariset, France) at a voltage window of 0.001–3 V (vs. Na/Na^+^) with a scan rate of 0.1 mV s^−1^. Galvanostatic charge/discharge profiles with cycle life and rate testing were conducted in the same voltage range as CV at different current densities using a BTS-610 battery tester (Neware, Shenzhen, China). Electrochemical impedance spectroscopy (EIS) was performed on Biologic VMP3 at a frequency range from 100 kHz to 10 mHz with an amplitude of 10 mV.

## 3. Results and Discussion

**Figure 2:**
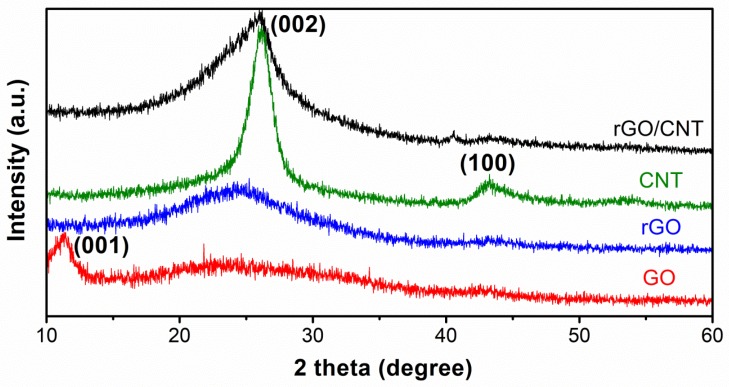
XRD scanned patterns of (a) rGO/CNT paper, (b) CNT, (c) rGO, and (d) GO, respectively.

XRD results of GO, rGO, CNT and rGO/CNT paper are plotted in Figure 2. The XRD curve of pristine GO exhibits a diffraction peak at 11.38° corresponding to (001) plane with a value of 0.78 nm as d-spacing. The distinctive GO peak disappears in the rGO pattern, indicating a reduction of the layered GO sheets. The rGO pattern shows a broad peak centered at 24.34° with a value of 0.37 nm as d-spacing, due to restoration of Van der Waals’ bonding interactions between rGO sheets upon reduction [41]. The acid-treated CNT exhibits two distinctive peaks at 43.02° and 26.3° corresponding to (100) and (002) planes with of values of 0.21 and 0.34 nm as d-spacings, respectively. (002) peak in rGO/CNT pattern slightly shifting position to lower 2θ of 25.22° indicates the interaction between rGO and CNT [42]. This phenomenon is attributed to CNT embedded in between rGO sheets inducing the slightly larger spacing in rGO/CNT [43].

**Figure 3:**
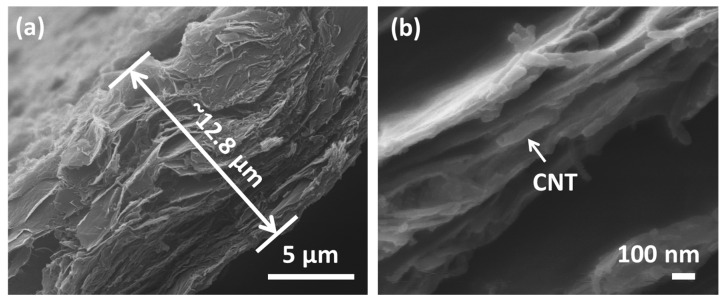
(**a**,**b**) SEM images of free-standing rGO/CNT paper cross-sectional views in different magnification.

The morphology of the hybrid flexible rGO/CNT paper was examined under SEM. Figure 3a shows a tilted view from the cross-section of paper under low magnification. The thickness of the paper is ≈ 12.8 µm. A uniformly distributed structure of the rGO/CNT layers with micron-sized wrinkles and pores can be observed. Figure 3b presents the tilted view of flexible carbon paper at higher magnification. It can be observed that there are CNTs in between rGO sheets to expand the distance of interlayers, which could potentially reduce the chance of rGO sheets stacking and enlarge the distance to facilitate easy diffusion of Na^+^ in the active electrodes.

**Figure 4:**
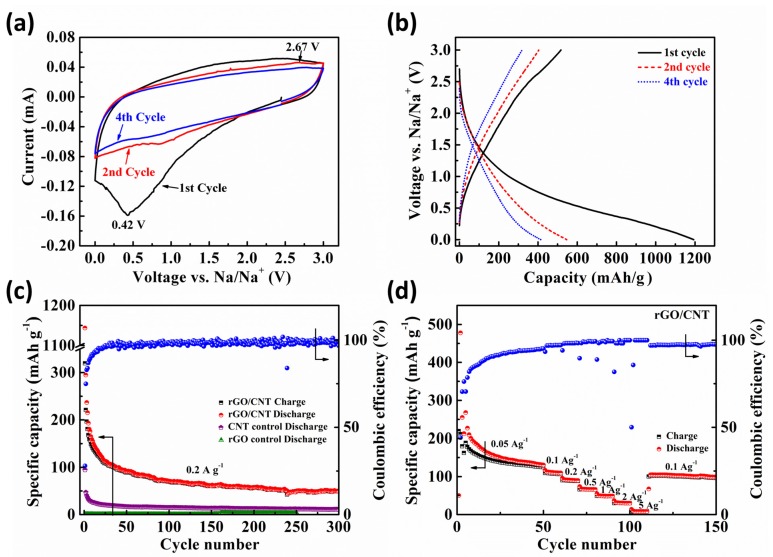
Electrochemical evaluation of free-standing rGO/CNT paper (**a**) Cyclic voltammograms in a voltage window from 0.001 to 3 V (vs. Na/Na^+^) with a scanning rate of 0.1 mV s^−1^, (**b**) Discharge/charge curves in the 1st, 2nd and 4th cycles at 0.1 A g^−1^, (**c**) cycle test results of free-standing rGO/CNT paper as well as two control samples rGO and CNT at a current rate of 0.2 A g^−1^ and (**d**) rate capability at various rates.

To study the electrochemical performance of free-standing rGO/CNTs paper as anode for NIBs, cyclic voltammetry (CV) was carried out and shows three representative CV curves of rGO/CNTs paper electrode in the voltage range from 0.001 to 3 V (vs. Na/Na^+^) are presented in Figure 4a. In the first cycle during the cathodic process, one main reduction peak can be observed at 0.42 V which is mainly due to the decomposition of electrolyte and solid electrolyte interface (SEI) layer formation [29]. This is an irreversible process and the peak disappeared during the subsequent cathodic scan. In the second cycle, a weak reduction peak can be observed at 0.75 V and assigned to the reaction between sodium ions and the functional groups on the carbon surface [18]. Both weak reduction peaks at approximated 0 V in the second and third cycle are attributed to the process of Na ions insertion into the rGO/CNT paper [15]. For the anodic scans, no readily apparent oxidation peaks can be observed, indicating that there is no specific voltage range for Na^+^ extraction from the active rGO/CNTs paper [34]. After the first cycle, the weak peaks and rectangular-shaped CV curves are indicative of a capacitive behavior of sodium storage. Meanwhile, the CV curves are almost overlapping with one the other, indicating acceptable reversibility and stability of free-standing electrode during the processes of Na^+^ insertion and extraction [18].

Figure 4b shows the galvanostatic discharge/charge performance of free-standing rGO/CNTs paper as NIBs anode at a current rate density of 0.1 A g^−1^. The first cycle of discharge/charge profile delivers initial specific discharge capacity of 1193.4 and charge capacity of 517.1 mAh g^−1^, respectively. The large loss of irreversible capacity is attributed to electrolyte decomposition as well as SEI layer formation, corresponding to the results of CV evaluation. In following cycles, the rGO/CNT electrode exhibits the sloping discharge/charge profiles. The possible sodium storage mechanism has been discussed from CV curves that the sloping voltage profiles are related to the sodium ion capacitive adsorption on rGO layers [44,45]. The majority of the storage capacity comes from the Na^+^ insertion on both surface sides of the rGO layers, which could provide abundant active accommodation sites for fast Na storage [18].

Figure 4c presents the cycling performance of free-standing rGO/CNT paper, control samples rGO and CNT at a current rate density of 0.2 A g^−1^. For rGO/CNT paper, there is capacity dropping at the initial ~ 30 cycles due to SEI film stabilization process, irreversible reaction process between Na^+^ and surface functional groups and Na^+^ insertion [18]. However, rGO/CNT electrode maintains a reversible specific capacity of 49.2 mAh g^−1^ at 250th cycle, which is higher than the capacity of 11.3 mAh g^−1^ for CNT and 2.6 mAh g^−1^ for rGO. After 30 cycles, coulombic efficiency approaches 100% and maintains the same at 300th cycle, indicating good capacity retention and cycling stability of the rGO/CNT electrodes. Rate capabilities of rGO/CNT paper were measured at various current densities to better understand the potential of using as flexible electrodes in NIBs. Figure 4d shows that the flexible electrode delivers average discharge capacities of 166.8, 111.3, 90.6, 67.3, 49.5, 31.1 and 9 mAh g^−1^ at various current rates of 50, 100, 200, 500, 1000, 2000 and 5000 mA g^−1^, respectively. After a deep cycling at a relatively high rate of 5000 mA g^−1^, when the density rate resets back to 100 mA g^−1^, the capacitiy can be recovered to 101.4 mAh g^−1^, indicating a good structural stability of electrode even cycling at a high current density. The interlayer distance of rGO is enlarged by CNT, which could potentially accelerate the transportation of Na^+^ in the hybrid electrode, leading to the enhancement of electrochemical performance. The rate capability performance of rGO/CNT paper is comparable to other studies on free-standing graphene anodes [7,46]. An et al. studied free-standing fluorine and nitrogen co-doped graphene paper as anode for flexible sodium-ion batteries [7]. When rate capability was performed at high current density of 1000 mA g^−1^, the electrode delivers a discharge capacity of 50 mAh g^−1^ which is pretty much the same as the capacity of our electrode in this work. Also compare to the work from David et al. [46], which is the study of thermally reduced graphene oxide paper as Na storage anodes, our electrode also showed better rate capacities compared to the capacities of ~ 20 mAh g^−1^ at high current rate for three types of rGO electrodes. Thus, the high rate capability performance demonstrates that the free-standing rGO/CNT paper in this work is a promising anode material for flexible NIBs.

**Figure 5:**
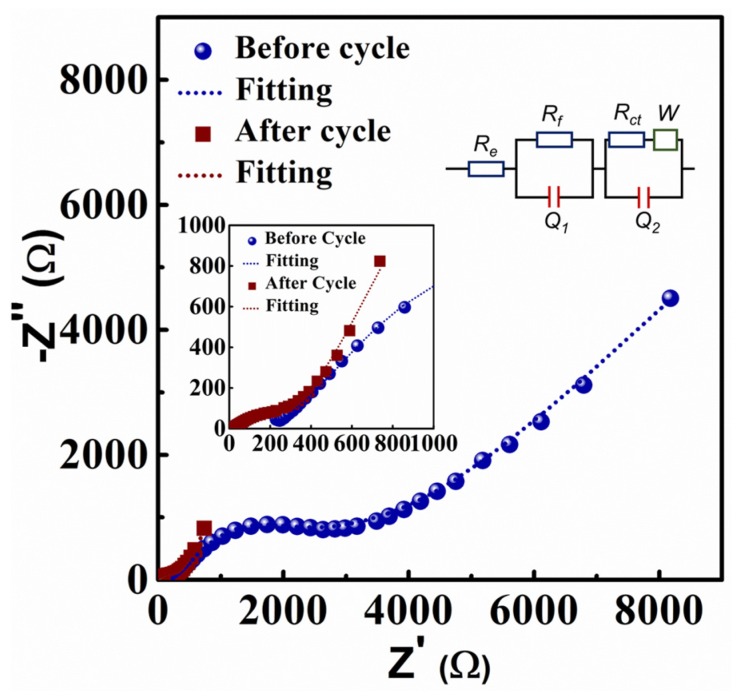
Nyquist plots of free-standing rGO/CNT paper in one Na–ion cell before and after cycle test. The insets show the semicircles in high frequency region and the equivalent circuit diagram used for fitting the Nyquist plots.

Electrochemical impedance spectroscopy (EIS) measurement was measured on the rGO/CNT electrode before cycle test and after 300 cycles. Figure 5 shows the diameter of the semicircle in high frequency in the Nyquist plots which represents the charge transfer resistance R_ct_. An equivalent circuit in Figure 5 inset is used to fit the Nyquist plots, where R_e_ denotes the electrolyte resistance, R_f_ denotes the interfacial resistance, R_ct_ denotes the charge transfer resistance, Q_1_ and Q_2_ represent related constant phase elements, and W is the Warburg element [47,48,49]. From the fitting results, rGO/CNT electrode before cycling exhibits a smaller charge transfer resistance R_ct_ of 12.26 Ω compared to the value of 964.1 Ω after cycling, indicating a higher charge transfer resistance after cycling. This could be due to the relatively higher polarization of sodium ions during cycling [44]. The interfacial resistance R_f_ before cycling is 383.5 Ω and shows much higher value of 2985 Ω after cycling, which could be mainly attributed to SEI layer resistance at the surface of electrode materials [50].

## 4. Conclusions

Free-standing and flexible reduced graphene oxide/carbon nanotube papers were successfully synthesized via an electrostatic spray deposition technique and electrochemically evaluated as anode material for flexible NIBs. The free-standing rGO/CNT paper possesses a capacitive charge storage, which contributes the sodium ion storage and stable cyclability. The rGO/CNT paper as flexible anode candidate for NIBs exhibits excellent rate performance with a specific capacity of 101.4 mAh g^−1^ at the current rate density of 0.1 A g^−1^, even after a high cycling rate of 5 A g^−1^. It can be concluded that the good electrochemical performance of this carbonaceous paper could be ascribed to the expansion of interlayer distance, facilitating transportation of sodium ions. Our study indicates free-standing rGO/CNT paper can be considered as a promising anode candidate for the flexible sodium-ion battery applications.
